# A touch-free human-robot collaborative surgical navigation robotic system based on hand gesture recognition

**DOI:** 10.3389/fnins.2023.1200576

**Published:** 2023-06-05

**Authors:** Jie Wang, Xinkang Zhang, Xinrong Chen, Zhijian Song

**Affiliations:** ^1^Academy for Engineering and Technology, Fudan University, Shanghai, China; ^2^Shanghai Key Laboratory of Medical Image Computing and Computer Assisted Intervention, Shanghai, China; ^3^Digital Medical Research Center, School of Basic Medical Science, Fudan University, Shanghai, China

**Keywords:** robot-assisted minimally invasive surgery, surgical robot, human-robot interaction, gesture recognition, contactless surgery

## Abstract

Robot-assisted minimally invasive surgery (RAMIS) has gained significant traction in clinical practice in recent years. However, most surgical robots rely on touch-based human-robot interaction (HRI), which increases the risk of bacterial diffusion. This risk is particularly concerning when surgeons must operate various equipment with their bare hands, necessitating repeated sterilization. Thus, achieving touch-free and precise manipulation with a surgical robot is challenging. To address this challenge, we propose a novel HRI interface based on gesture recognition, leveraging hand-keypoint regression and hand-shape reconstruction methods. By encoding the 21 keypoints from the recognized hand gesture, the robot can successfully perform the corresponding action according to predefined rules, which enables the robot to perform fine-tuning of surgical instruments without the need for physical contact with the surgeon. We evaluated the surgical applicability of the proposed system through both phantom and cadaver studies. In the phantom experiment, the average needle tip location error was 0.51  mm, and the mean angle error was 0.34 degrees. In the simulated nasopharyngeal carcinoma biopsy experiment, the needle insertion error was 0.16  mm, and the angle error was 0.10 degrees. These results indicate that the proposed system achieves clinically acceptable accuracy and can assist surgeons in performing contactless surgery with hand gesture interaction.

## 1. Introduction

Robot-assisted minimally invasive surgery (RAMIS) is now well established in clinical practice due to its high precision and minimal invasiveness (Nagyné Elek and Haidegger, 2019; Haidegger et al., 2022). In RAMIS, preoperative medical image data is utilized to plan the surgical path, while the robot performs the approach during the surgery as per the plan. Surgeons must manipulate various software to control the navigational surgical robot throughout the procedure, especially to fine-tune surgical instruments with their perspective in complex surgeries. However, at present, adapting the surgical robot by manual means increases the risk of bacterial diffusion, rendering the surgeon unable to control the robot during surgery while complying with the high sterile requirements. To address this issue, various types of study have been proposed. Some studies have attempted to solve this problem by using other devices such as joysticks and pedals to transform the surgeon’s command into actions (Díaz et al., 2014; Ohmura et al., 2018). Nevertheless, in the case of joysticks, human-robot interaction (HRI) tasks applied to surgical robots are performed through master–slave operations, with which has not been effectively resolved on the movement difference between the master and slave console and the problem of over-operation. On the other hand, the pedal-based solutions are still limited by behavioral consistency, which impedes their use for every surgeon in RAMIS, particularly those who are unskilled. Recently, several studies have attempted to address this issue through contactless HRI using touch-free solutions (Nestorov et al., 2016; Cho et al., 2018; Despinoy et al., 2018), with a particular focus on hand gesture recognition-based HRI. The researchers have made significant progress in modeling and analyzing hand gesture recognition. These studies have adopted various frameworks to predict users’ intentions in HRI tasks and enable robots to perform corresponding actions, including probabilistic graphical models of temporal processes, deep learning techniques with supervised learning, and other methods including unsupervised learning algorithms, among others (Van Amsterdam et al., 2021; Cao et al., 2022).

Probabilistic graphical models of temporal processes, which have been widely utilized in speech recognition for time series analysis, have also served as a source of inspiration for gesture recognition in HRI tasks (Ahmidi et al., 2017). Chen et al. (2015) introduces a novel hand gesture recognition model based on hidden Markov models (HMM), which could identify a worker’s gesture patterns and intentions with reliable accuracy. Mavroudi et al. (2018) proposes a framework for fine-grained gesture segmentation and recognition, which employs a Conditional Random Field (CRF) model and a frame-level representation based on discriminative sparse coding. Reiley et al. (2008) utilizes Linear Discriminant Analysis (LDA) and HMM to build models for gesture recognition, which improved the recognition rate by promoting discrimination between sub-gestures instead of the entire gesture, thus enabling them to capture the internal variability of each segment. The aforementioned models have been implemented effectively to analyze the kinematic signals for the da Vinci surgical robot. Deep learning techniques, specifically the implementation of deep convolutional neural networks (CNN), have been employed for the purpose of recognizing gestures. In the study by Oyedotun and Khashman (2017), the image is first preprocessed using binarization, followed by setting a threshold to locate the gesture, and finally, a CNN is utilized to recognize the gestures. Similarly, ElBadawy et al. (2017) uses a 3D CNN-based gesture recognition system to analyze normalized images, achieving a recognition rate averaging 90%. Huynhnguyen and Buy (2021) introduces a 2-stage surgical gesture recognition approach, where one stage detects the transition between consecutive gestures using a 3D CNN, and the other stage classifies video clips into corresponding gesture classes based on a long short-term memory (LSTM) neural network. Experimental results using JIGSAWS’s suturing video dataset show that the proposed method achieves an accuracy of over 70% for both tasks. Moreover, Fang et al. (2019) presents a gesture recognition system that combines generative adversarial network (GAN) and CNN, achieving better results with fewer samples.

Furthermore, there are alternative approaches for gesture recognition in HRI tasks. Huang et al. (2011) presents a gesture recognition approach that relies on Gabor filters and a support vector machine (SVM) classifier. Their proposed method is highly resistant to variations in illumination, leading to recognition rates that improve from 72.8 to 93.7%. Tarvekar (2018) introduces a skin threshold segmentation approach for recognizing and categorizing gestures by segmenting hand regions in images and extracting color and edge features through an SVM classifier. Shi et al. (2021) proposes a novel domain adaptive framework for robotic gesture recognition that aligns unsupervised kinematic visual data, enabling the real robot to acquire multi-modality knowledge from a simulator. The empirical evidence indicates that the model has the potential to significantly enhance the operational efficiency of the real robot, resulting in a noteworthy 12.91% increase in precision. Moreover, there exist cases in which the recognition of hand gestures is facilitated through the utilization of Leap Motion™ and Kinect™ devices (Ahmad et al., 2016; Jin et al., 2016).

In current RAMIS procedures, limited interactions between the surgeon and the robot restrict surgical efficiency. And it is apparent that the majority of present-day studies employ relatively intricate techniques to achieve specific HRI tasks using various devices. However, little study has presented a comprehensive framework for gesture recognition that exhibits strong generalization capabilities and high efficiency, which can be applied effectively to address the problem of the contactless HRI in RAMIS. To this end, we propose a concise and effective framework for navigational surgical robots to perform actions in response to the surgeon’s gestures in this paper, utilizing touch-free solutions based on hand gesture recognition technology. This framework facilitates the robot to execute surgical interventions under the guidance of an expert surgeon and a surgical navigation system, resulting in enhanced medical treatment efficacy and conserved healthcare resources, while also ensuring aseptic conditions that impede bacterial dissemination.

## 2. Materials and methods

### 2.1. System composition

**Figure 1:**
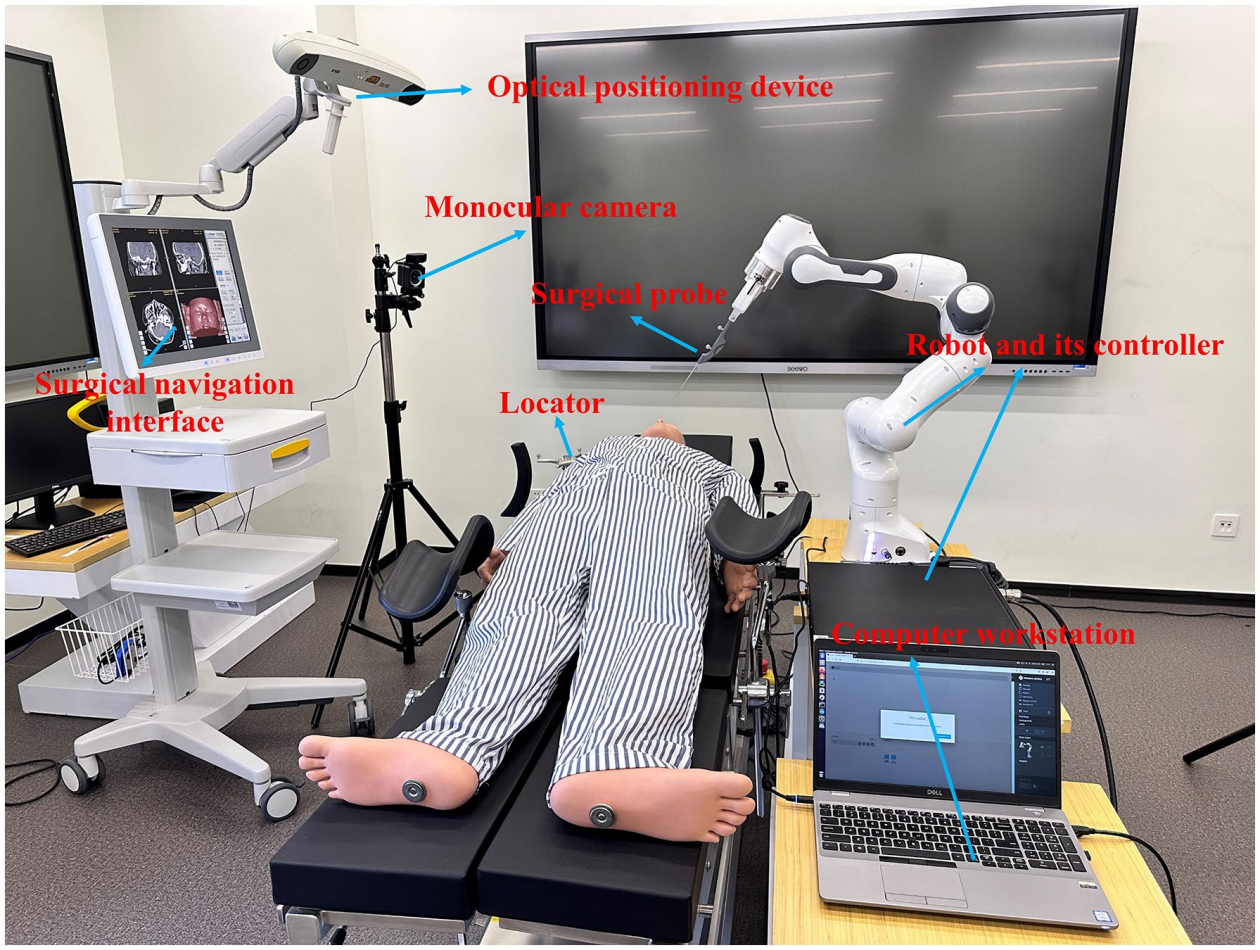
Overview of the non-contact collaborative surgical navigation robot system.

As depicted in Figure 1, the collaborative surgical navigation robot system is primarily composed of a computer workstation, an optical positioning-based surgical navigator, auxiliary accessories such as surgical probes and locators, and a robot module that includes a 7-DoF robotic arm and its controller. Both the operating table and surgical navigator are mobile devices that can be adjusted to fit the patient and surgeon’s positions. The workstation and its internal software connect the surgical navigator and the robot into a closed-loop structure. The surgical navigator tracks the patient and surgical instruments in real-time by positioning reflective balls mounted on the operating table and the robot manipulator. The collaborative surgical robot, with its terminal surgical instruments, can be positioned flexibly around the operation table and controlled by the surgeon’s hand gestures. The navigator constructs an enhanced surgical field by integrating preoperative medical information of patients (e.g., target organs, vessels and planned surgical paths) with the location and target points of intraoperative instruments attached to the actual patient body to provide surgeons with augmented visual information. With the direct navigation interface, hand gesture guidance can be used as a direct and natural method to interact with the surgical robot.

### 2.2. System workflow

**Figure 2:**
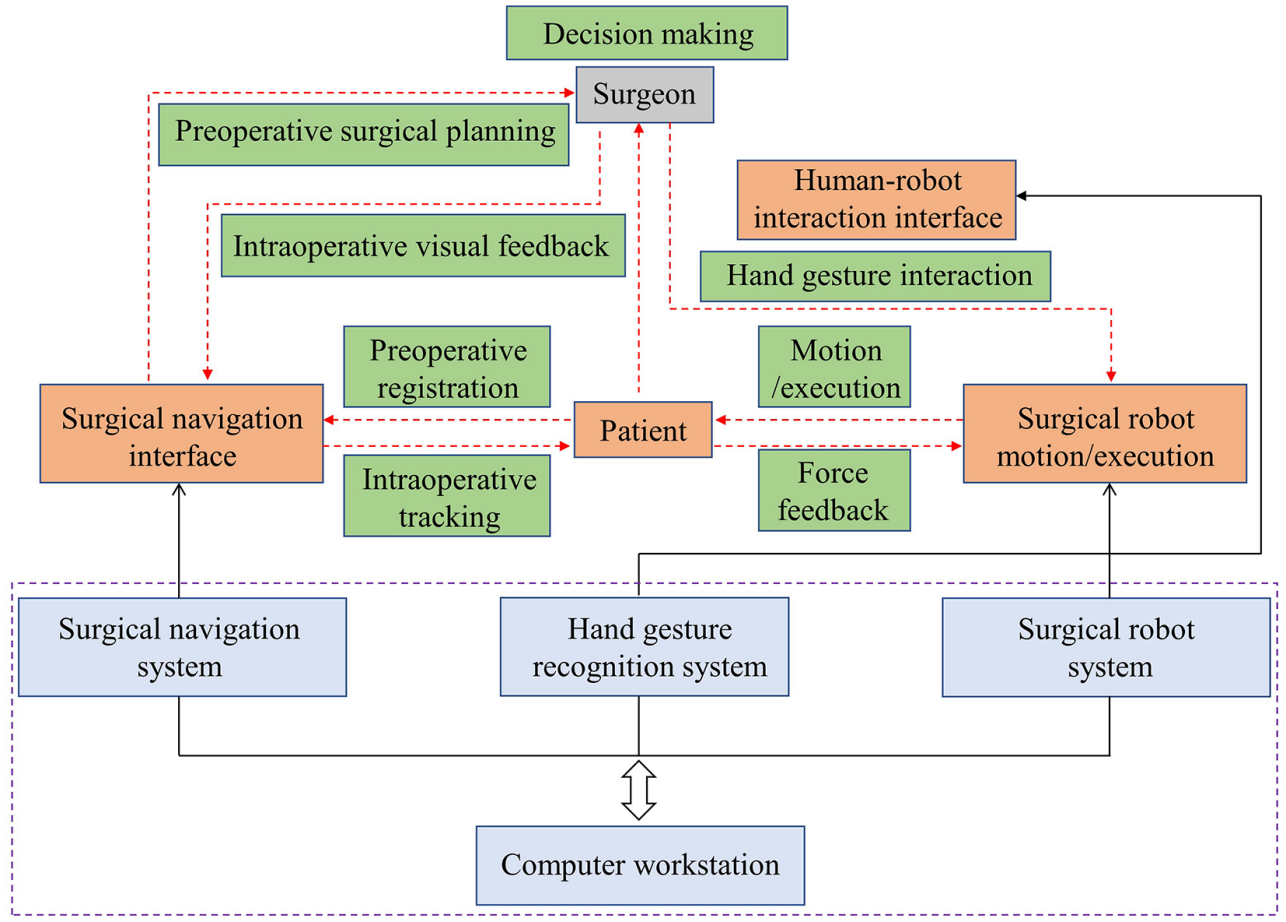
Workflow of the hand-gesture based surgeon-robot cooperation.

The figure displayed in Figure 2 outlines the workflow of the proposed surgical system. The computer workstation serves as the main control and computation center, enabling robot control, generating enhanced surgical visual information, and supporting human-robot interaction. The hardware layer of the collaborative surgical robot system is denoted with blue dotted lines, while the human subjects involved in the touch-free surgical procedure, such as the surgeon, surgical navigation interface, surgical robot motion/execution, patient, and HRI interface, are located above this blue layer. The data flow is indicated by black arrow lines, including control and feedback flows between the subjects and hardware modules. The surgeon-centered interaction flow is shown with red dotted lines, highlighting the data flow among the subjects. For this contactless robot-assisted puncture treatment with a surgical navigation interface, a semi-automatic mode of surgical procedure is proposed. Surgeons are required to select surgical targets and needle insertion sites through patient image guidance before surgery and plan corresponding surgical paths. During surgery, surgeons can fine-tune the needle’s posture directly through hand gesture interaction. The generation of the surgical navigation interface is based on our previous research (Liu et al., 2017; Chen et al., 2021). This paper centers on the attainment of contactless HRI objectives, specifically, the detection of gestures and the subsequent control of surgical robots.

### 2.3. Gesture recognition model

**Figure 3:**
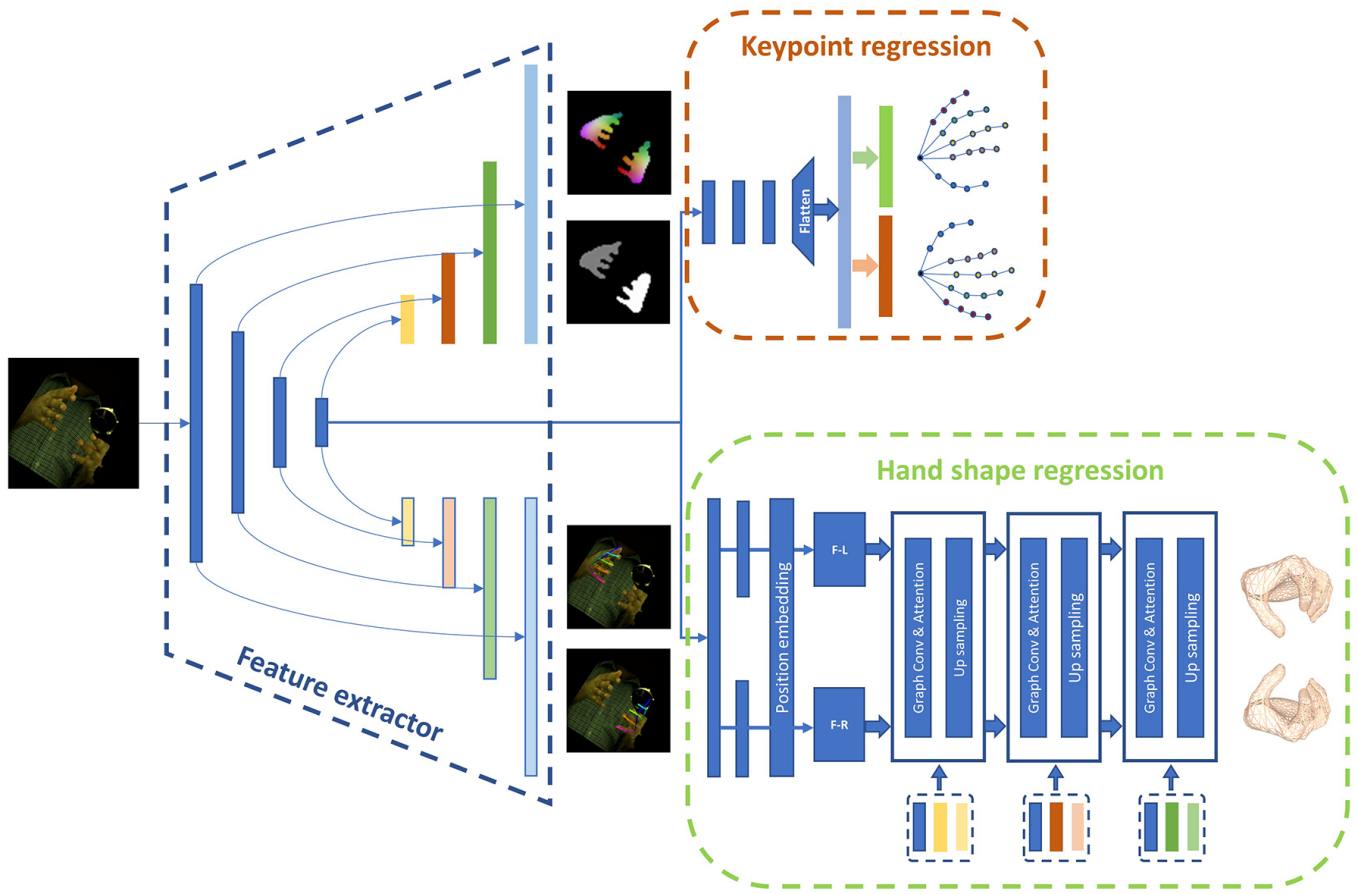
The concrete architecture of the hand gesture recognition model.

In this section, our attention is directed towards the gesture recognition module of our approach. The specific architecture of the model is illustrated in Figure 3.

The main function of the hand gesture recognition network is to process monocular images captured by the camera to acquire the desired pose and shape of the hand. The 3D pose of the hand is denoted by the 3D position of keypoints, while the shape of the hand is represented in the form of a mesh. We have identified a total of 21 keypoints on the hand as regression targets, which include the position of the wrist, finger joints, and fingertips. The 3D position of each key point is denoted by the (x, y, z) coordinates. The hand shape is represented by a mesh consisting of 778 nodes, with associated connection information between them. We represent the mesh in the network as a graph G(V, E), where V represents the 778 nodes and E denotes the connection information between them. Our gesture recognition module employs a Unet architecture and utilizes a multi-layer convolutional network for feature extraction, resulting in a feature map of varying sizes. This is followed by convolution and upsampling to extend the feature map and combine it with the previously extracted features at each layer. Our approach is divided into several distinct parts.

#### 2.3.1. Keypoint regression branch

In order to simultaneously regress the pose of both hands, a regression approach is utilized to predict the keypoint locations. The position information of the hand keypoints, with a shape of (2, 21, 3), is unfolded into a 126-dimensional vector, which is then paired with dataset labels to calculate the L2 loss values. This enables the network to learn how to regress the keypoints. Eq. (1) depicts the generation of the keypoint positions. The regression process employs the last layer of the encoder output, which passes through multiple homogeneity networks and is subsequently expanded into a 126-dimensional vector that represents the 3D positions of joints.


(1) $$P=f_{flat}\left(f_{de}\left(f_{en}\left(X_{img}\right)\right)\right)$$

where $P\in {\mathbb{R}}^{2\times 21\times 3}$ indicates the vector of 126 dimensions, $X_{img}$ is the RGB image, $f_{en}$ and $f_{de}$ represent encoder and decoder of the regression branch respectively, and $f_{flat}$ denotes a flatten function that can convert a three-dimensional matrix to a 126-dimensional vector.

#### 2.3.2. Auxiliary prediction branch

Three distinct auxiliary methods are employed to aid the model in making accurate predictions. These methods consist of hand segmentation, density mapping, and 2D pose. The 2D pose branch transforms the ultimate feature map of the Unet architecture into 21 heatmaps, which denote the 2D positions of both hands. Meanwhile, the hand segmentation branch restores the feature map to its original resolution, producing a mask with distinct pixel values for the left hand, right hand, and background. In addition, dense matching produces a dense mapping map with the same dimensions as the original map by establishing correspondences between images in a manner analogous to positional coding. We utilize dense matching to define the correspondence between vertices and image pixels, employing various hues to represent individual vertices.

The three categories of auxiliary information are labeled independently in the dataset and are utilized to compute the loss values, allowing the network to more effectively extract hand features.

#### 2.3.3. Hand shape regression branch

Convolutional mesh regression is utilized in this branch to generate precise and dense 3D shapes for the hands. This classical method produces a 3D mesh aligned with the image, enabling the generation of intricate and fine 3D shapes. The hand shape regression branch comprises a network with graph convolution. As demonstrated in Eq. 2, the feature maps are spanned and propagated into two fully connected layers with a position embedding module to derive the left-hand and right-hand graph structures, respectively. The process for the former graph structure is illustrated in Eq. 3, where it is first subjected to graph convolution and subsequently, the outputs are passed through a multi-head attention mechanism module to establish attention between nodes within itself and merge with the features extracted from different layers by the feature extractor. Finally, it is transmitted to the interaction attention module across the left and right hands to determine the interaction relationship between the hands and assist in modifying their shape information. The hand mesh is generated in a coarse-to-fine approach, where a coarse mesh is initially generated, and then, according to the nearest neighbor mode, it is up-sampled following the rules of graph coarsening to acquire a finer mesh, with the features of the coarse mesh assigned to its children vertices. With the final layer of the graph processed, a mesh consisting of 778 vertices is obtained.


(2) $$V_{L}^{0},V_{R}^{0}=f_{g}\left(F_{img}\right)$$

where $F_{img}$ represents the feature map from Resnet50 encoder, $f_{g}$ indicates the function that converts feature map into graph structure with fully connected layers and position embedding module.


(3) $$V_{L}^{i+1},\;V_{R}^{i+1}=G_{i}\left(\left[V_{L}^{i},\;V_{R}^{i},\;I_{i}\right]\right),\;i=0,1,2$$

where $V_{L}^{i}$ and $V_{R}^{i}$denote the hand vertices of the i-th layer of the left and right hand shape regression branch, respectively. $I_{i}$ is the feature map from the encoder and decoder, $G_{i}$ indicates the function with Graph convolution, interaction attention, and up-sampling.

### 2.4. Hand gesture mapping to robot

Upon identifying hand gestures, it becomes imperative to regulate the surgical robot’s motion, ensuring the meticulous adjustment of surgical instruments, culminating in the seamless execution of a non-invasive surgical procedure. To maneuver the robot with precision, it is essential to encode the hand’s posture and correspondingly map it to appropriate commands.

#### 2.4.1. Encoding

To quantify the position information of keypoints, we initially need to extract appropriate features. We opt to use the Euclidean distance between joints to calculate the distance between each pair of keypoints. By using features with high differentiation, we can accurately represent and distinguish various commands, thereby enhancing the system’s reliability. For this purpose, we employ the distance between the fingertip and the root point as features for binary encoding, where a distance greater than a predefined threshold is encoded as 1 and vice versa. The binary encoding principle is illustrated in Eq. 4.


(4) $$B_{i}=\left\{\begin{array}{l} 1\;\;\mathrm{if}\;d_{i-0}\geq threshold\\ 0\;\;\mathrm{otherwise} \end{array}\right.$$

where $d_{i-0}$ indicates the distance between the i-th finger’s tip and root point, and $B_{i}$ represents the code for the corresponding finger.

#### 2.4.2. Gesture mapping

**Figure 4:**
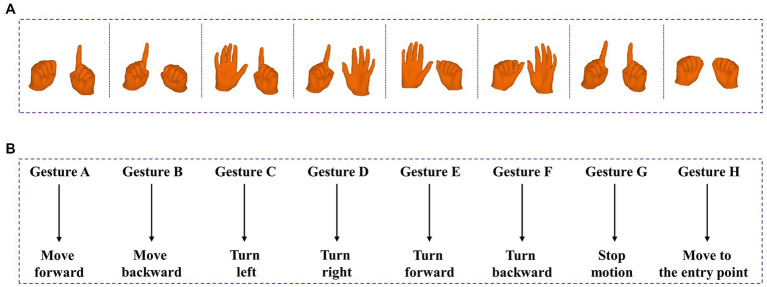
Predefined 8 gestures and its corresponding commands in robot. **(A)** From left to right are: Gesture A, Gesture B, Gesture C, Gesture D, Gesture E, Gesture F, Gesture G and Gesture H. **(B)** Gesture-motion corresponding rules.

To ensure optimal system stability and minimize the risk of accidental touches, a dual-hand posture control method is employed. A set of eight distinctive hand gestures has been carefully selected to showcase this control scheme, as illustrated in Figure 4. Gesture A involves clenching the left hand while extending the forefinger of the right hand. In Gesture B, the right hand is clenched while the left hand extends the forefinger. Gesture C is characterized by an open left hand with the right hand extending the forefinger, while in Gesture D, the right hand is open with the left hand extending the forefinger. Gesture E entails an open left hand while the right hand is clenched, and Gesture F involves an open right hand while the left hand is clenched. In Gesture G, both hands extend their forefingers, whereas in Gesture H, both hands are clenched.

#### 2.4.3. Safety strategies for HRI

**Figure 5:**
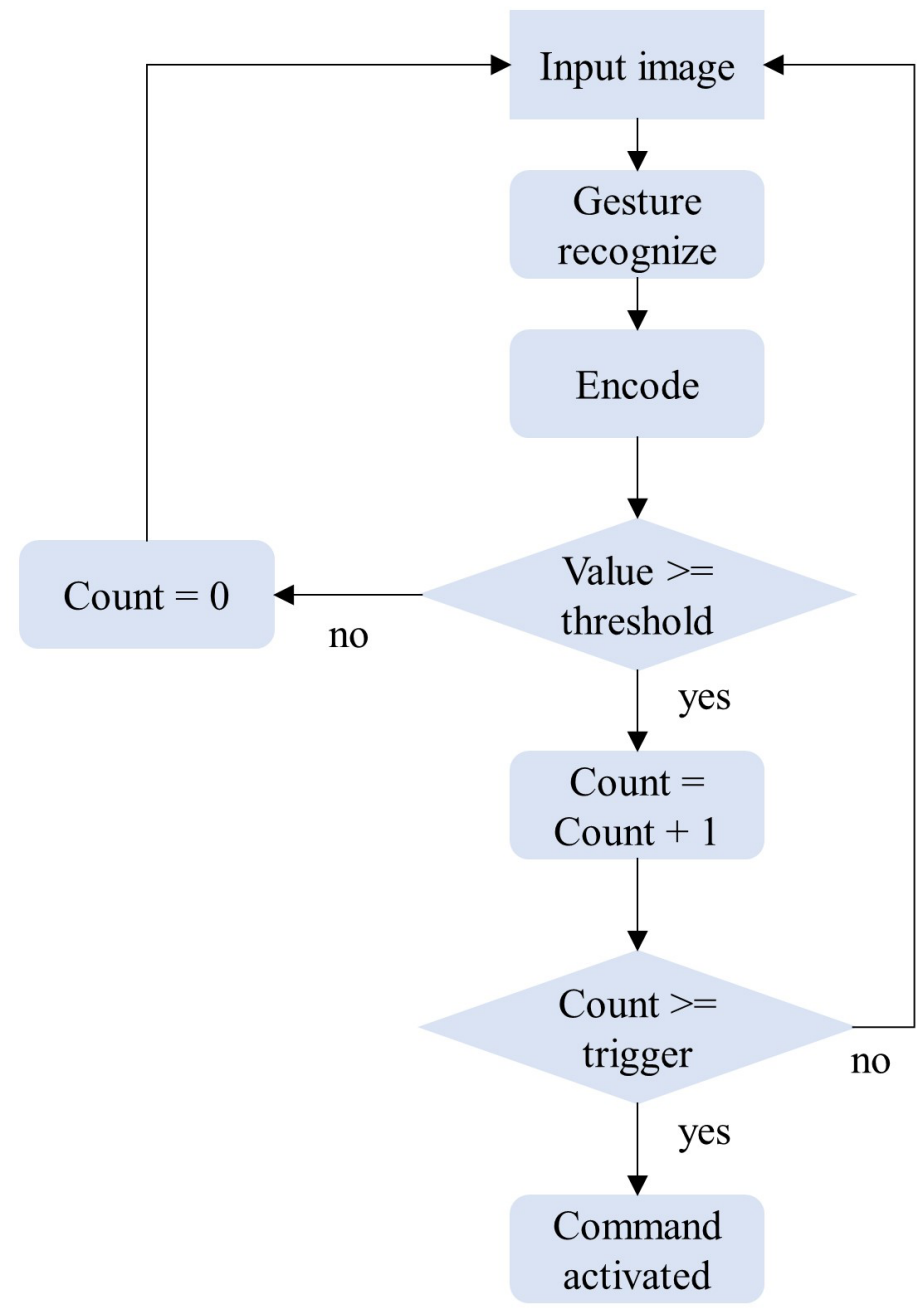
State machine design for HRI tasks.

Our methodology involves employing a continuous and uninterrupted stream of video frames that are captured by the camera. Relying on a single frame for recognition and command transmission would give rise to ambiguity and instability within the system. To circumvent this, we formulated a simple state machine to manage and filter the triggers of gestures. As shown in Figure 5, we assigned a distinct counter for each gesture, which increments each time the recognition outcomes correspond to the code of that specific gesture and resets to zero if another gesture appears. To curtail erroneous touches and bolster the robustness of the system, we programmed the counter to activate the corresponding control command when it reaches a specific threshold. Following the triggering of a gesture, the counter does not immediately clear but rather remains at a value above the threshold until a subsequent action clears it.

## 3. Results

The hand gesture interaction model is implemented on the collaborative robotic arm, Franka Emikia, and its effectiveness is verified through experiments on phantom and cadaver research, following the process outlined in Figure 2.

### 3.1. Gesture recognition accuracy

In order to apply gesture recognition model to surgical robots, it is necessary to first test the accuracy of recognizing predefined gestures. We evaluated the recognition accuracy and corresponding robot operation effects of eight gestures through two experiments involving 10 volunteers. In the first experiment, each volunteer performed the predefined gestures at different locations, and each gesture was tested five times on the same volunteer. The average recognition accuracy for each gesture is shown in Table 1. It is worth noting that there was one recognition failure in the third and fifth categories, which was due to the fingers being obstructed. In the second experiment, volunteers manipulated the robot through gestures to complete a specified task, aimed at verifying the learning difficulty and efficiency of the gesture interaction. The completion time for the task, which involved touching a specified object, ranged from 1 min 27 s to 2 min 32 s among the 10 volunteers, with an average of 1 min 49 s. These results demonstrate that the gestures we designed for interaction are straightforward and easily learned, and that the corresponding actions of the robot are reasonable.

**Table 1:** Accuracy of eight gesture recognition with predefined categories.

Category	1	2	3	4	5	6	7	8
Accuracy	100%	100%	98%	100%	98%	100%	100%	100%

### 3.2. Phantom experiment

#### 3.2.1. Experimental settings

The phantom experiment involved the use of a surgical robot to perform expected actions based on human hand gestures on a skull model. A target tumor composed of a metal nail with dimensions of 2 × 2 × 2 mm was prepared in the eyebrow center of a phantom to simulate the location of the puncture target. Simultaneously, we located a metal block, measuring 4 mm^3^, onto the model’s nasal tip to imitate the surgical entry point. The skull model was then subjected to CT scanning and introduced into a surgical navigator to simulate surgical path planning, with the entry point being the tip of the nose and the target location being the eyebrow center.

**Figure 6:**
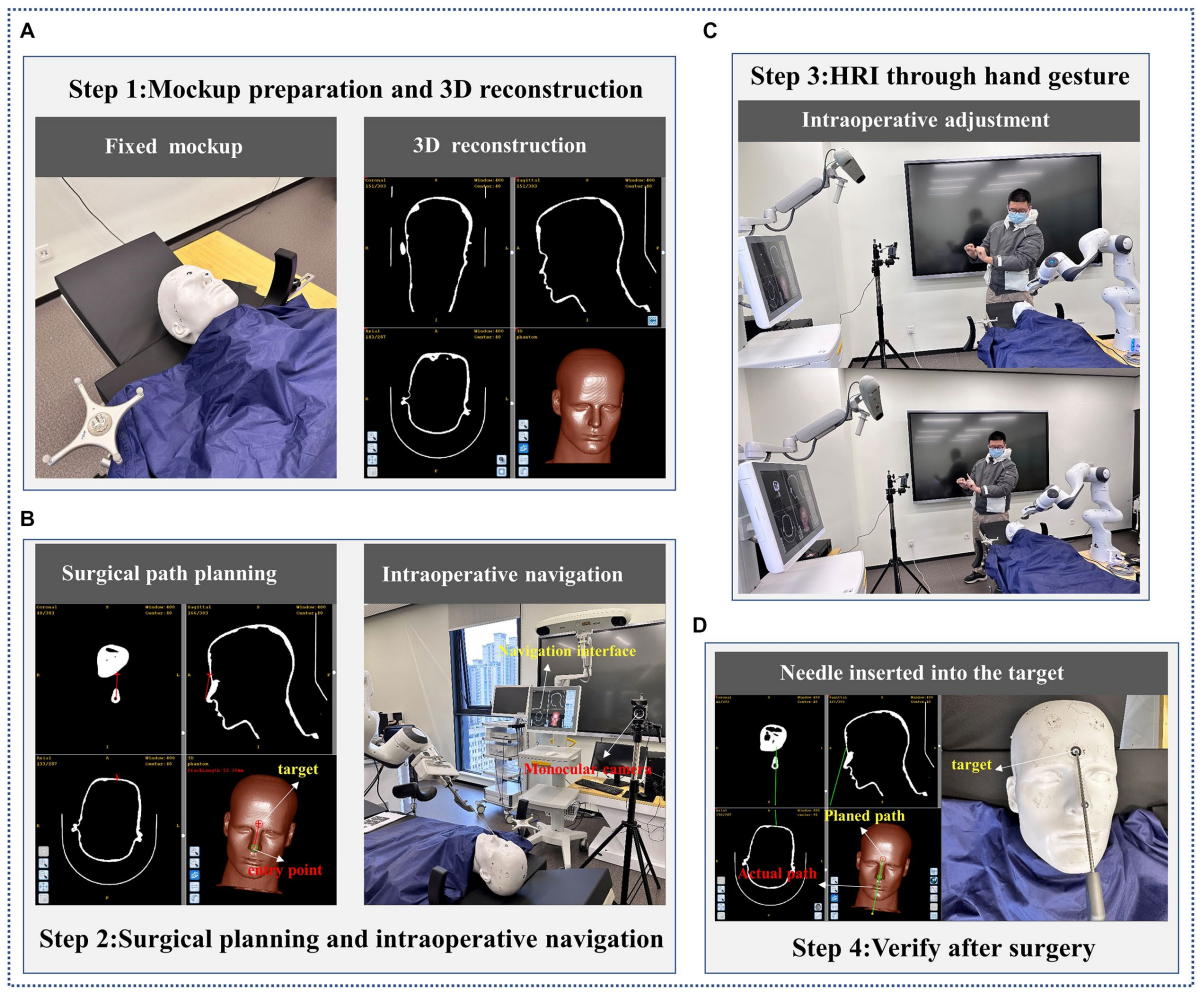
The flow of phantom experiment. **(A)** Mockup preparation and 3D reconstruction; **(B)** Surgical planning and intraoperative navigation; **(C)** HRI through hand gesture; **(D)** Verify after surgery.

A surgeon from a hospital participated in the phantom experiment. With the guidance of the surgical navigation system, the needle held by the surgical robot was gradually inserted through touch-free hand gesture interaction with the surgeon. The target and actual path of the needle were derived after insertion into the phantom, and the position error of the needle tip in the preoperative plan was estimated with the help of the surgical system. The workflow of the phantom experiment is illustrated in Figure 6.

#### 3.2.2. Experimental result

Table 2 showcases the mean positioning error of the needle tip and the rotation angle error of the needle. The experimental data reveals that the needle tip’s average positioning error is 0.51 mm, and the average angle error is 0.34 degrees. The results obtained from the surgeon’s five experiments are outlined in Table 2.

**Table 2:** Error of the needle insertion in phantom experiment.

Number of experiments	Needle	
	Position error (mm)	Angle error (°)
1	0.40	0.14
2	0.54	0.22
3	0.67	0.57
4	0.63	0.41
5	0.32	0.36

### 3.3. Cadaver experiment

#### 3.3.1. Experimental settings

**Figure 7:**
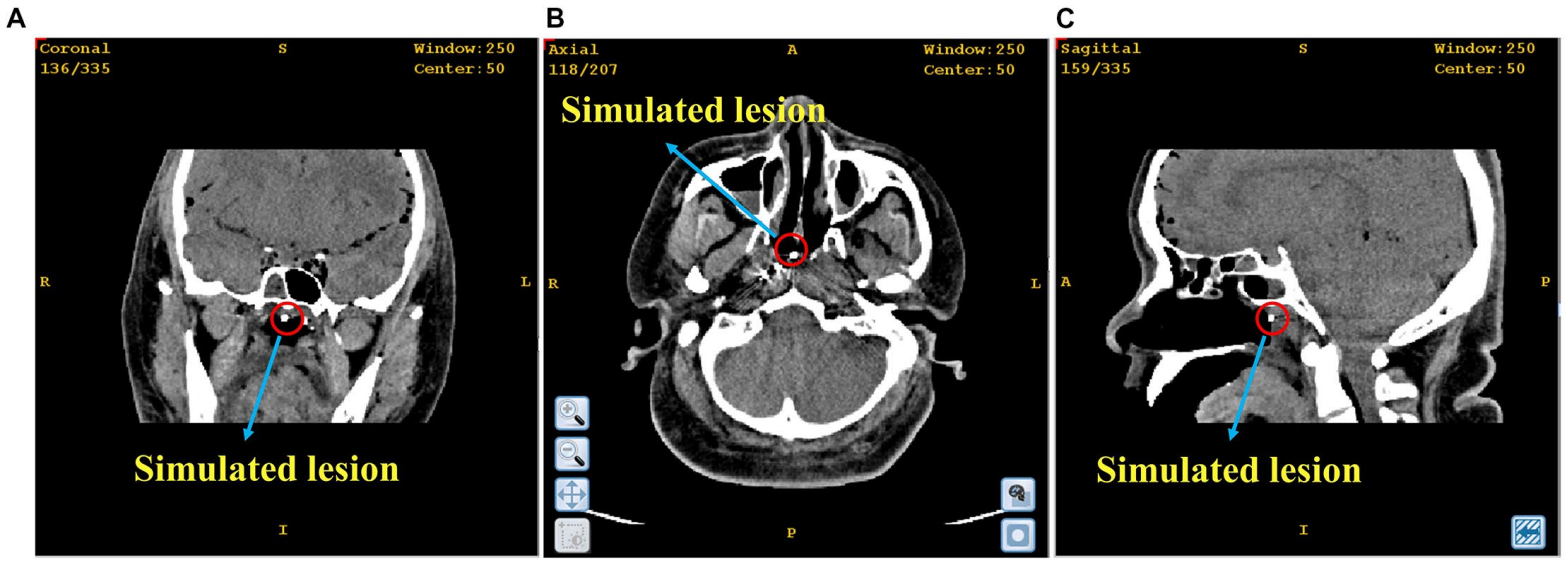
The position of the simulated lesion in nasopharyngeal apex. **(A)** Coronal plane; **(B)** Cross section; **(C)** Sagittal plane.

In this section, a simulated experiment was conducted to perform a biopsy for nasopharyngeal carcinoma on a cadaver. To create the lesion, we placed a small metal block with a volume of 8 mm^3^ at the nasopharyngeal apex of the cadaver head. CT scanning was then performed to obtain medical information with marked points of the cadaver, as illustrated in Figure 7. Using these CT data, we performed 3D reconstruction to create an image-guided space where surgical planning could take place. The needle entry point was located at the top of the anterior nostril, and the target point was at the top of the nasopharynx where the metal block was located.

Similar to the phantom experiment, the surgical robot adjusted its position gradually until the needle tip reached the insertion point of the surgical path, following the hand gesture interactive instructions of the surgeon. With the guidance of surgical navigation and hand gesture interaction, the needle was positioned to align with the planned path and maintained in that posture until it reached the lesion. In the same way, we estimated the errors in the location and angle of the needle tip.

#### 3.3.2. Experimental result

**Figure 8:**
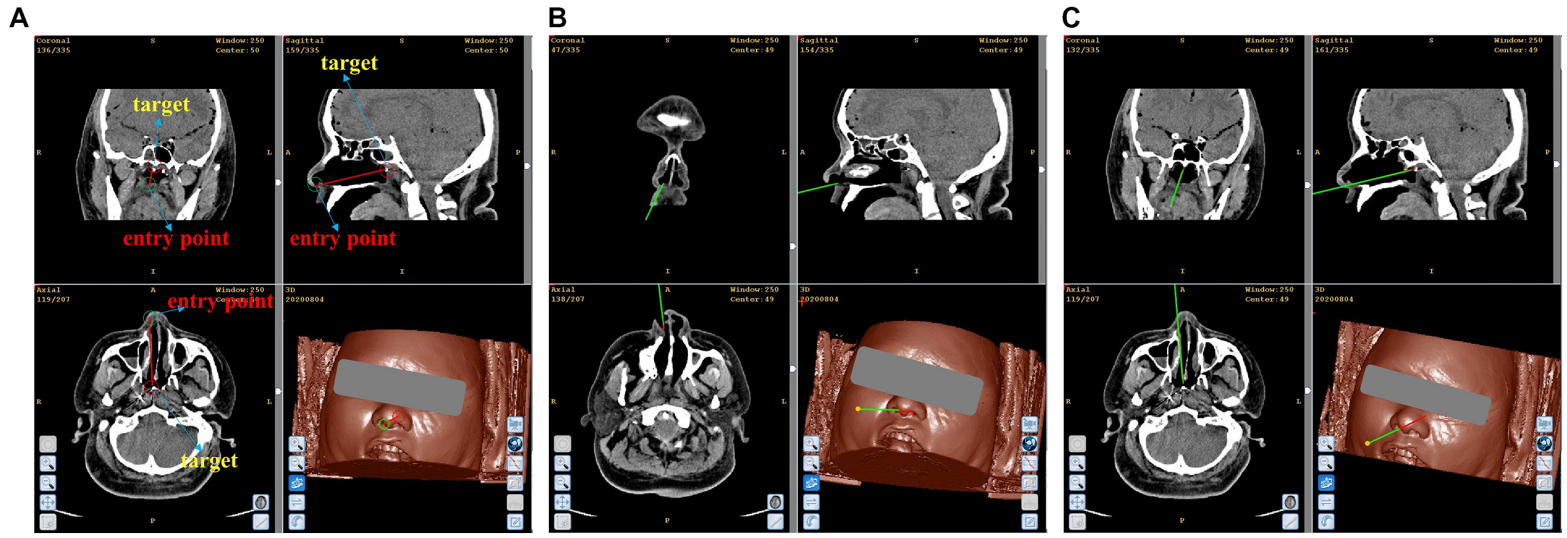
Simulated biopsy results. **(A)** Simulated surgical path. **(B)** The needle reaches the top of the anterior nostril. **(C)** The needle reaches the simulated lesion.

Figure 8 illustrates the outcomes of the simulated biopsy for nasopharyngeal carcinoma. The red and green lines displayed in the figure indicate the intended surgical path and the real needle location, respectively. In Figure 8A, the path of the simulated biopsy is demonstrated. Figure 8B exhibits that the needle approached the entry point with a posture that is in agreement with the planned path. Ultimately, Figure 8C portrays the outcome of the needle insertion into the simulated lesion. As can be seen from Figure 8, the actual needle position nearly matches the planned surgical path. By fitting the needle information in the surgical navigator, we obtained an actual location error of 0.16 mm and an angle error of 0.10 degrees.

## 4. Discussion

In this study, we presented a novel framework for recognizing gestures using monocular color images, achieving an accuracy rate of over 98% in recognizing all predefined gestures. Compared with traditional manual procedures, the proposed framework for gesture recognition facilitates efficient contactless interaction between surgeons and surgical robot in RAMIS, thereby mitigating the risk of bacterial transmission and enhancing surgical efficacy by enabling precise fine-tuning of related instruments.

Both phantom experiments and cadaver studies were successfully conducted to provide proof of concept for the contactless HRI to assist in RAMIS, and it is evident that sub-millimeter precision was achieved after implementing the trials with hand gesture interaction. We suggest two potential rationales for the positive results observed in our experimentation. The first evidence of the enhanced precision in hand gesture recognition is derived from the auxiliary prediction branch, which significantly contributes to the extraction of both 3D and 2D hand features. Another possible explanation could be that each adjustment of the robot we designed is considerably subtle, especially in terms of its ability to make adjustment for orientation. This, in turn, increases the possibility of accurate movement of the surgical robot according to the intended surgical plan. Moreover, the surgical robot can be configured with high efficiency, and the HRI interface exhibits a shallow learning curve (the average learning time of only 1 min and 49 s) in a simulated task, thereby results in no significant increase in surgery preparation time. It was proved that with the aid of hand gesture interaction, the surgeon can effortlessly fine-tune surgical instruments without physical contact for a majority of the time.

Although the current version of the non-contact collaborative surgical navigation robot system has showed favorable outcomes, it is not without its limitations. One example of this is the limitation faced by the surgeons in adjusting the surgical instruments, as they can merely adjust them through one gesture at a time. This results in an increase of the motion steps and a decrease in task efficiency. We think this can be effectively solved by designing more gestures. Additionally, the complex environment of the operating room with a multitude of medical instruments and limited space may lead to restricted image acquisition and occasional hand occlusion, resulting in recognition failures. Hand occlusion is also the reason for the two cases of recognition failure in Table 1. Furthermore, the proposed state machine may cause discomfort as it necessitates maintaining a gesture for a period of time, while the designed model lacks recognition of dynamic gestures, limiting the surgeon’s control over the surgical robots through dynamic gestures.

At present, the available data from phantom and cadaver cases is sufficient to establish the feasibility of the touchless HRI interface for RAMIS. We believe that our work will be regarded as the fundamental basis of touchless surgical robot HRI, and it has been preliminary substantiated by both phantom and cadaveric investigations. The findings have indicated the efficacy of the design of the collaborative system in aiding other surgical procedures involved RAMIS and demand stringent sterility standards. We believe that the framework we have established will form a practical system and be applied in clinic.

## 5. Conclusion

The feasibility and validity of the framework we proposed in this paper are verified through the experiments on both phantoms and cadavers. The experimental findings evince the surgical robot’s ability of fine-tuning instruments through augmented visual feedback from the navigation surgical system and contactless hand gesture recognition, thus by minimizing bacterial, the surgical safety can be enhanced. At the same time, the framework is easily integrated into a real surgical robot. The future works should endeavor the study of surgical robot application utilizing mixed reality technology that integrates touch-free solutions and the development of more dynamic hand gestures to augment the integration flexibility.

## Data availability statement

The original contributions presented in the study are included in the article/supplementary material, further inquiries can be directed to the corresponding authors.

## Author contributions

JW organized and performed experiments, wrote and revised the manuscript. XZ proposed and did the research and designed the model. XC and ZS conceived the initial idea, supervised the work, and revised the manuscript. All authors contributed to the article and approved the submitted version.

## Funding

This work was supported by the National Natural Science Foundation of China (Grant No. 62076070) and the Local Cooperation Project of artificial Intelligence Medical Hospital in Xuhui District, Shanghai (Grant No. 2021–008).

## Conflict of interest

The authors declare that the research was conducted in the absence of any commercial or financial relationships that could be construed as a potential conflict of interest.

## Publisher’s note

All claims expressed in this article are solely those of the authors and do not necessarily represent those of their affiliated organizations, or those of the publisher, the editors and the reviewers. Any product that may be evaluated in this article, or claim that may be made by its manufacturer, is not guaranteed or endorsed by the publisher.
